# Supplementary material for: Major agricultural changes required to mitigate phosphorus losses under climate change
Source: Nat Commun. 2017 Jul 31;8:161. doi: 10.1038/s41467-017-00232-0 (PMC5534432; doi:10.1038/s41467-017-00232-0)
Supplement: Supplementary file 1 — Supplementary Information [file 41467_2017_232_MOESM1_ESM.pdf]

File name: Supplementary Information
Description: Supplementary Figures, Supplementary Tables and Supplementary References

File name: Supplementary Data 1
Description: Discharge and total phosphorus load predicted by 'Hydrological Predictions for the Environment' (HYPE) and 'Data-Based Mechanistic' (DBM) models for the future

File name: Supplementary Data 2
Description: Total phosphorus loads predicted by 'Hydrological Predictions for the Environment' (HYPE) model for combined land management change (-80%, -50%, -20%, no change, +20%, +50%, +80% change in P inputs) and climate change scenarios for the Eden, Wensum and Avon Demonstration Test Catchments.

File name: Supplementary Data 3
Description: Hydrological Predictions for the Environment (HYPE) model: Parameter ranges used in HYPE behavioural runs.

File name: Supplementary Data 4
Description: Hydrological Predictions for the Environment (HYPE) model fit statistics

File name: Supplementary Data 5
Description: Data-Based Mechanistic model (DBM) fit statistics

File name: Peer Review File
Description:

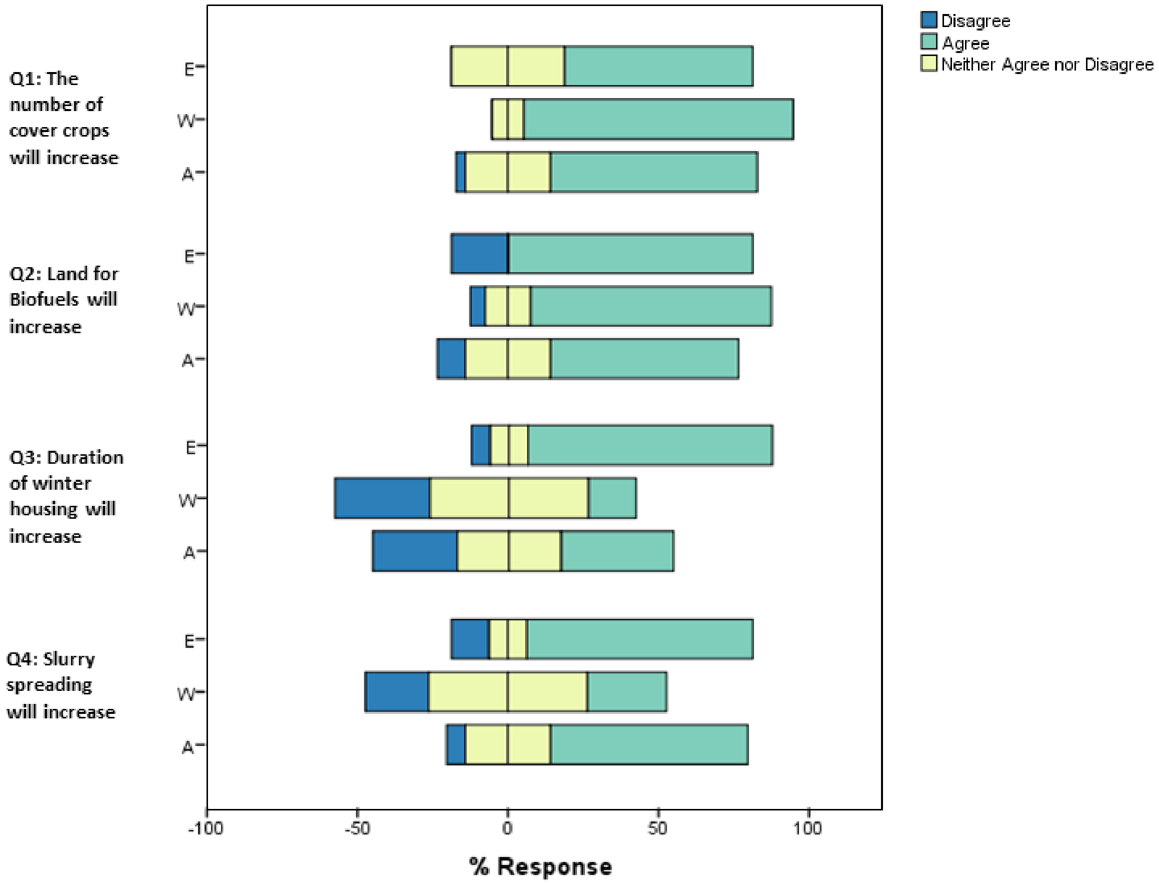

Supplementary Figure 1 Stakeholder responses to the four questions (Q1 – Q4) on mitigation options. Responses were grouped as positive (strongly agree or agree), neutral (neither agree nor disagree) or negative (disagree or strongly disagree). Workshop locations were Eden (E), Wensum (W) and Hampshire Avon (A). Numbers of participants were n(E)=16; n(W)=20; n(A)=32.

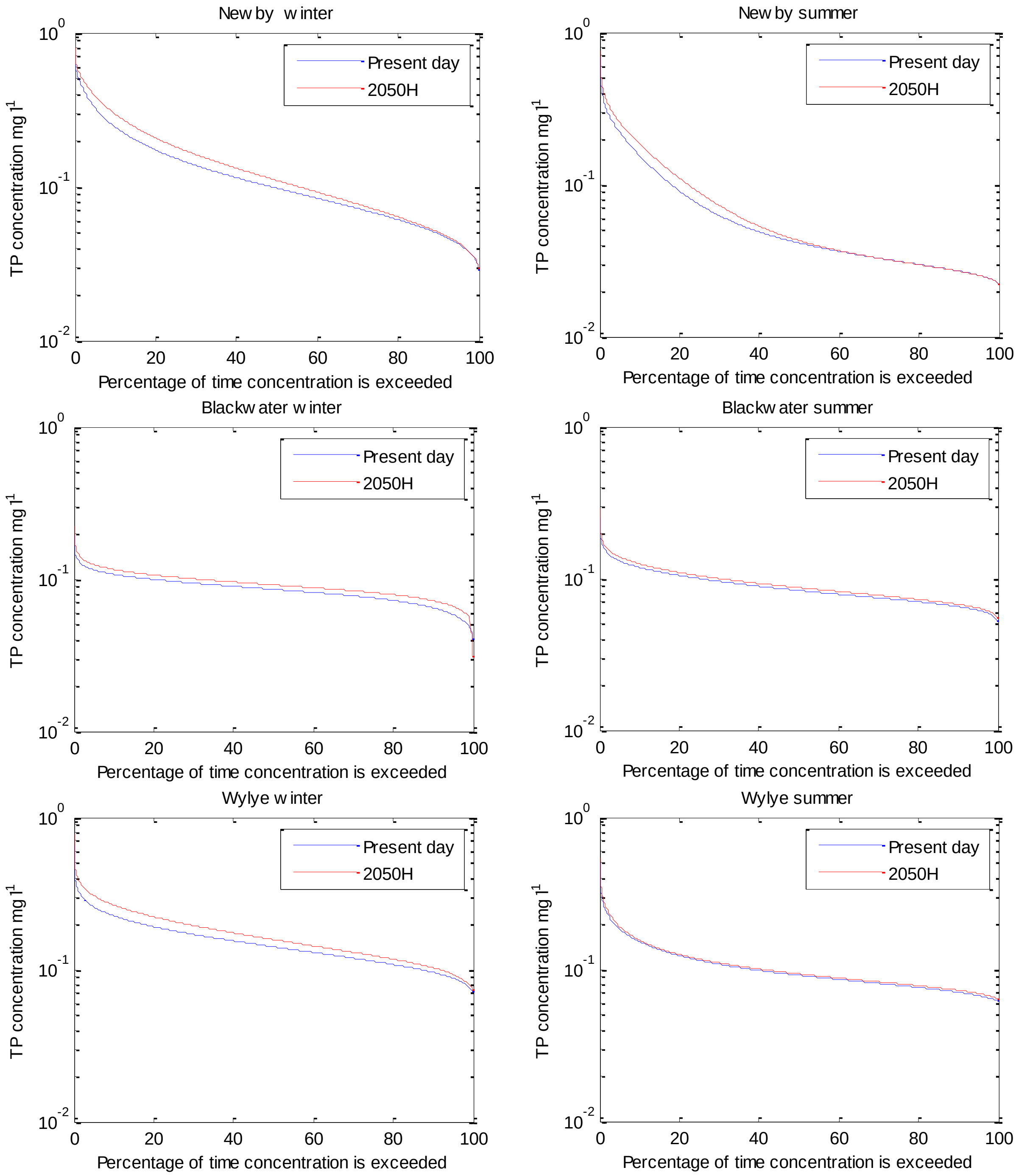

Supplementary Figure 2 Winter and summer concentration-duration curves predicted with HYPE for each catchment; Newby Beck, Eden, winter and summer (top); Blackwater, Wensum, winter and summer (middle); Wylye, Avon, winter and summer (bottom).

Supplementary Table 1 Model structures and parameters for Data-Based Mechanistic (DBM) models used in simulations

Site	Model output	Model input	Model structure	β	a1	a2	b1	b2
Newby, Eden	Discharge Q	Rainfall R	Continuous [2, 2, 1]	0.37	0.3474 ± 0.0064	0.0023 ± 0.0001	0.1646 ± 0.0026	0.0026 ± 0.0001
Newby, Eden	Total P load TP	Effective rainfall Re	Continuous [1, 1, 1]		0.6429 ± 0.0191		2.0086 ± 0.0562	
Blackwater, Wensum	Discharge Q	Rainfall R	Discrete [2, 2, 6]	0.65	-1.9324 ± 0.0021	0.9325 ± 0.0021	0.0526 ± 0.0012	-0.0521 ± 0.0012
Blackwater, Wensum	Total P load TP	Rainfall R	Continuous [2, 2, 4]		0.0826 ± 0.0018	0.00021 ± 0.00003	0.0335 ± 0.0012	0.00016 ± 0.00002
Wylye, Avon	Discharge Q	Rainfall R	Discrete [2, 2, 6]	0.59	-1.7785 ± 0.0109	0.7790 ± 0.0108	0.0440 ± 0.0016	-0.0428 ± 0.0015
Wylye, Avon	Total P load TP	Effective rainfall Re	Continuous [2, 2, 6]		0.1660 ± 0.0080	0.00029 ± 0.00003	1.3015 ± 0.0506	0.0054 ± 0.0006

Supplementary Table 2 Differences between RCM 1.5 km climate model and UKCP09-WG, as used in this study

	1.5 km Regional Climate Model (RCM 1.5 km)	UKCP09 Weather Generator (UKCP09-WG)
Source model	1.5 km UKV regional climate model (driven by 12km and 60km climate models)	11 member HadRM3 ensemble, 25km resolution.
Bias correction	Not bias corrected (see footnote)*	UKCP09-WG has little bias as it is fitted to the 5 x 5 km gridded rainfall observations (1961-1990)
Emissions scenarios	RCP8.5	SRES Storylines Low (b1), Medium (a1b), High (a1fi)
Periods	Baseline (1996-2009), 13 years ~2100.	Baseline (1961-1990), 2050s (2040-2069), 2080s (2070-2099); 100 randomly generated runs of 30 years for each
Data resolution	1.5 km	Point-based (no spatial coherence between adjacent 5 km grid squares)
Advantages	Convection permitting model allows more realistic representation of rainfall extremes at local scale.<br>Large number of variables available.	Feasible to produce large numbers of scenarios for uncertainty analysis<br>Point-based time series based on statistics of local weather
Disadvantages	Large data volumes only permit relatively short run lengths, limiting ability to capture extreme events in the time series.<br>Too computationally demanding to generate large ensembles of runs for uncertainty analysis.	Projections not spatially coherent<br>Statistical model cannot capture physical mechanisms.<br>Limited number of variables available.

* Previous work using bias corrected climate data and an ensemble of hydrological and biome models as part of the Inter-Sectoral Impact Model Intercomparison Project (ISI-MIP)[1] has shown that although the bias correction largely removes the impact of differences between GCMs in the present-day baseline data, there is still large variability due to the different impact models and the processes included; for instance, the effect of CO_2 on runoff added to the uncertainty in model projections.

Supplementary Table 3 Metadata for generation of UKCP09 Weather Generator time series data for Newby Beck, Cumbria. Emissions scenarios are from SRES storylines: b1 (Low); a1b (Medium); a1fi (High).

	Newby Beck at Newby, Eden, Cumbria					
Dataset	wxgen					
WGVersionNumber	2.1.0					
DataOutputFormat	csv					
SpatialAverage	grid_box_5km					
Location	3650525; 3600525; 3600520; 3650520					
SamplingMethod	random					
WGTemporalFrequency	hourly					
NumberOfRandomSamples	100					
WGRunDuration	30					
WGRandomSeedSwitch	True	True	True	True	True	True
EmissionsScenarios	b1	a1b	a1fi	b1	a1b	a1fi
TimePeriods	2040-2069			2070-2099		
WGRandomSeedValue	2014	2015	2016	2083	2081	2082

Supplementary Table 4 Metadata for generation of UKCP09 Weather Generator time series data for Blackwater, Wensum. Emissions scenarios are from SRES storylines: b1 (Low); a1b (Medium); a1fi (High).

	Blackwater at Park Farm, Wensum, East Anglia					
Dataset	wxgen					
WGVersionNumber	2.1.0					
DataOutputFormat	csv					
SpatialAverage	grid_box_5km					
Location	6150325; 6150330; 6100330; 6100325					
SamplingMethod	random					
WGTemporalFrequency	hourly					
NumberOfRandomSamples	100					
WGRunDuration	30					
WGRandomSeedSwitch	True	True	True	True	True	True
EmissionsScenarios	b1	a1b	a1fi	b1	a1b	a1fi
TimePeriods	2040-2069			2070-2099		
WGRandomSeedValue	2001	2002	2003	2004	2005	2006

Supplementary Table 5 Metadata for generation of UKCP09 Weather Generator time series data for Wylye, Avon. Emissions scenarios are from SRES storylines: b1 (Low); a1b (Medium); a1fi (High).

	Wylye at Brixton Deverill, Avon, Hampshire					
Dataset	wxgen					
WGVersionNumber	2.1.0					
DataOutputFormat	csv					
SpatialAverage	grid_box_5km					
Location	3800140; 3850140; 3900140					
SamplingMethod	random					
WGTemporalFrequency	hourly					
NumberOfRandomSamples	100					
WGRunDuration	30					
WGRandomSeedSwitch	True	True	True	True	True	True
EmissionsScenarios	b1	a1b	a1fi	b1	a1b	a1fi
TimePeriods	2040-2069			2070-2099		
WGRandomSeedValue	2020	2021	2022	2085	2086	2087

Supplementary Table 6 Discussion questions for stakeholder workshops on agricultural change and interpretation for modelling

Discussion questions for the Eden (the same questions (apart from catchment name) were asked in the Wensum and Avon catchments)<br><br>1. How do you think percentages of land use class will alter in the Eden in the future?<br><br>2. How do you think crop production will change in the Eden in the future?<br><br>3. How do you think livestock production will change in the Eden in the future?

Supplementary Table 7 Questionnaire completed by stakeholder workshop participants in Eden catchment. The same questionnaire (apart from catchment name) was completed in Wensum and Avon catchments.

Land Use and Land Management scenarios Eden 5th April 2016

What is your role in water quality management?

- Farmer/land manager ☐
- Farm advisor ☐
- Policy advisor ☐
- Regulator ☐
- Academic ☐
- Other (please specify) ☐

If you have a particular catchment association (Morland/Pow/Eden), please answer the remaining questions with that catchment in mind. Which catchment are you answering for?

- Morland/Pow ☐
- Eden ☐
- General ☐

How important do you consider the following factors to be in contributing to land use and land management changes?

	Very Important	Important	Neutral	Unimportant	Very Unimportant
Climate change	☐	☐	☐	☐	☐
Economics	☐	☐	☐	☐	☐
Policy	☐	☐	☐	☐	☐
Legislation	☐	☐	☐	☐	☐
Technology	☐	☐	☐	☐	☐
Other (please specify)					

Overall land use

Studies on regional land use change scenarios indicate that, in some areas, the percentage of land used for food production will decrease (global food production will be concentrated in the most productive areas, and near regions of highest population increase, with increased food demand more than balanced by improvements in technology and crop yields). Land formerly used for food production may be replaced by woodland or biofuels or abandoned

	Strongly Agree	Agree	Neither agree nor disagree	Disagree	Strongly Disagree
Land for crop production will increase	☐	☐	☐	☐	☐
Land for livestock production will increase	☐	☐	☐	☐	☐
Land for forestry will increase	☐	☐	☐	☐	☐
Land for biofuels will increase	☐	☐	☐	☐	☐
Other comments					

Crop production
Higher seasonal rainfall may make some crops increasingly unviable
Higher temperatures may extend the growing season, making different crops more viable
Incentives for water quality management may be improved (CAP greening)

	Strongly Agree	Agree	Neither agree nor disagree	Disagree	Strongly Disagree
Maize production will increase	☐	☐	☐	☐	☐
Other cereals (for food) will increase (please specify)	☐	☐	☐	☐	☐
The number of different crops will increase	☐	☐	☐	☐	☐
Cover crops will increase	☐	☐	☐	☐	☐
Other comments					

Livestock production
Higher temperatures may extend the growing season
Regulations on slurry spreading are likely to become tighter
Dairy herds are likely to get fewer but larger, to maintain viability

	Strongly Agree	Agree	Neither agree nor disagree	Disagree	Strongly Disagree
Livestock density in the catchment will increase	☐	☐	☐	☐	☐
Capacity for winter housing will increase	☐	☐	☐	☐	☐
Duration of winter housing will increase (livestock in earlier/out later)	☐	☐	☐	☐	☐
Slurry spreading (total amount) will increase	☐	☐	☐	☐	☐
Covered yards will become more common	☐	☐	☐	☐	☐
Other comments					

Thank you!

Supplementary Reference

1. Davie JCS, *et al.* Comparing projections of future changes in runoff from hydrological and biome models in ISI-MIP. *Earth Syst Dynam* **4**, 359-374 (2013).
